# Draft Genome Sequence of Mycobacterium sp. Strain JC1 DSM 3803

**DOI:** 10.1128/MRA.00150-21

**Published:** 2021-05-13

**Authors:** Mitchell G. Thompson, Christopher B. Eiben, Allison N. Pearson, Patrick M. Shih

**Affiliations:** a Feedstocks Division, Joint BioEnergy Institute, Emeryville, California, USA; b Biological Systems & Engineering Division, Lawrence Berkeley National Laboratory, Berkeley, California, USA; c Department of Plant Biology, University of California, Davis, Davis, California, USA; d Perlumi Chemicals, Inc., Berkeley, California, USA; e Genome Center, University of California, Davis, Davis, California, USA; f Environmental Genomics and Systems Biology Division, Lawrence Berkeley National Laboratory, Berkeley, California, USA; University of Maryland School of Medicine

## Abstract

Mycobacterium sp. strain JC1 DSM 3803 is one of the few known bacteria predicted to possess the xylulose monophosphate (XuMP) pathway of C_1_ assimilation. The draft genome is 7,921,603 bp with a GC content of 66.88% and will allow more in-depth investigation of this bacterium’s unique metabolism.

## Abstract (precis)

Mycobacterium sp. strain JC1 DSM3803 is one of the few known bacteria predicted to possess the xylulose monophosphate (XuMP) pathway of C1 carbon assimilation. The draft genome is 7,921,603 bp with a GC content of 66.88% and will allow more in depth investigation of the bacterium’s unique metabolisms.

## ANNOUNCEMENT

Mycobacterium sp. strain JC1 DSM 3803 has piqued the interest of researchers due to the diversity of C_1_ assimilation pathways it employs. Isolated from soil in Seoul, South Korea, the bacterium was originally misclassified as an Acinetobacter sp. before 16S rRNA sequencing showed it to be a Mycobacterium sp. (1). Subsequent work has revealed that the bacterium does not appear to employ the canonical prokaryotic ribulose monophosphate (RuMP) cycle (2), but it possesses functional ribulose bisphosphate (RuBP) (2, 3) and xylulose monophosphate (XuMP) cycles (4). Although some short fragments of the gene clusters responsible for these carbon assimilation pathways have been sequenced, the lack of a full-genome sequence prevents a complete understanding of this organism’s metabolic potential.

We obtained Mycobacterium sp. JC1 DSM 3803 from the DSMZ-German Collection of Microorganisms and Cell Cultures (DSM 3803) and passaged pure cultures once before isolating the genomic DNA. To prepare the genomic DNA, 10 ml of bacterial culture was first grown overnight in brain heart infusion medium (BD Biosciences, San Jose, CA, USA) at 30°C, from which 1 ml was pelleted and stored at −80°C. Genomic DNA was isolated via phenol-chloroform extraction followed by ethanol precipitation, as described previously (3). Illumina library preparation and sequencing were performed by the Vincent J. Coates Genomics Sequencing Laboratory. Genomic DNA was fragmented using a Covaris M220 sonicator and size selected using AMPure XP beads to isolate fragments of 300 bp. Libraries were prepared using library preparation kits from Kapa Biosystems (Wilmington, MA, USA) and sequenced with a 150-bp paired-end NovaSeq S4 flow cell (Illumina, Inc., San Diego, CA, USA). Paired-end reads were then checked for quality with FastQC 0.11.9 (4) and trimmed using Trimmomatic 0.36 with the settings LEADING:30 TRAILING:30 MINLEN:120, resulting in 54,496,719 surviving read pairs (5). The genome was assembled *de novo* using SPades version 3.10.1 (6), and the assembly quality was assessed with QUAST 5.0.2 (7). The assembly resulted in 72 contigs over 2,000 bp (*N*_50_, 224,424 bp; *L*_50_, 10), comprising a genome with a total size of 7,921,603 bp, a GC content of 66.88%, and an average read coverage of 1,365×. Contigs were annotated via the Prokaryotic Genome Annotation Pipeline version 5.1 (8). Unless otherwise stated, all software was run using default settings.

The PGAP annotation pipeline predicts that Mycobacterium sp. JC1 DSM 3803 contains 7,493 coding sequences, of which 1,127 are annotated as hypothetical proteins (8, 9). Interestingly, while previous work could not detect any RuMP activity (2), there are homologs of both 3-hexulose-6-phosphate synthase (JNN96_36860) and 6-phospho-3-hexuloisomerase (JNN96_36865) in the genome, suggesting that the bacterium may be able to utilize the RuMP pathway under certain conditions. Further work dissecting the complex metabolism of this bacterium may reveal the reasons for such a diversity of methylotrophic pathways in a single organism.

### Data availability.

This whole-genome sequencing project has been deposited in NCBI GenBank under the accession no. PRJNA694986, and the Illumina short-read data have been deposited in the SRA under the accession no. SRR13553746.
